# Genome Sequence of a Novel Kunsagivirus (*Picornaviridae*: *Kunsagivirus*) from a Wild Baboon (*Papio cynocephalus*)

**DOI:** 10.1128/genomeA.00261-17

**Published:** 2017-05-04

**Authors:** Connor R. Buechler, Adam L. Bailey, Michael Lauck, Anna Heffron, Joshua C. Johnson, Cristine Campos Lawson, Jeffrey Rogers, Jens H. Kuhn, David H. O’Connor

**Affiliations:** a Department of Pathology and Laboratory Medicine, University of Wisconsin-Madison, Madison, Wisconsin, USA; b Wisconsin National Primate Research Center, Madison, Wisconsin, USA; c Human Genome Sequencing Center, Baylor College of Medicine, Houston, Texas, USA; d Integrated Research Facility at Fort Detrick, National Institute of Allergy and Infectious Diseases, National Institutes of Health, Fort Detrick, Frederick, Maryland, USA

## Abstract

The picornaviral genus *Kunsagivirus* has a single member, kunsagivirus A, which was discovered in migratory bird feces. We report here the discovery of a novel kunsagivirus in wild yellow baboon (*Papio cynocephalus*) blood. The genomic sequence of this virus indicates the probable need for the establishment of a second kunsagivirus species.

## GENOME ANNOUNCEMENT

The *Picornaviridae* family of the order *Picornavirales* contains viruses with positive-sense single-stranded RNA genomes that produce nonenveloped virions. Picornaviruses infect birds, fish, and mammals belonging to a diverse array of species, including primates. Currently, the family consists of 54 species grouped into 31 officially recognized genera, including the recently formed genus *Kunsagivirus*. Kunsagivirus A (strain Roller/SZAL6-KuV/2011/HUN, GenBank accession number KC935379) is the only classified member of the only species included in the genus, *Kunsagivirus A*. This virus was discovered in a fecal sample collected in Hungary in July 2011 from an Afro-Palearctic long-distance migratory bird, the European roller (*Coracias garrulus*), using sequence-independent random reverse transcriptase PCR (RT-PCR) amplification of virion-associated nucleic acids, 5′/3′ rapid amplification of cDNA ends (RACE), and Sanger sequencing (1). However, as this virus was found in the feces of only a single bird, it is unclear whether Kunsagivirus A naturally infects roller birds or a food source.

Here, we report the genomic sequence of a novel virus detected in the blood of baboon M27, a wild adult male yellow baboon (*Papio cynocephalus*) sampled in Mikumi National Park in Tanzania in 1986 (2). In brief, RNA was isolated from blood plasma using the MinElute virus spin kit without carrier RNA (Qiagen, Valencia, CA), and random hexamers were used to prime cDNA synthesis (Life Technologies, Inc., Grand Island, NY), as previously described (3). Deep-sequencing libraries were prepared using the Nextera XT kit (Illumina, San Diego, CA) and sequenced on an Illumina MiSeq. Low-quality (Phred <Q30) and short reads (<100 bp) were removed with CLC Genomics Workbench 7.1 (CLC bio, Aarhus, Denmark), and the remaining reads were assembled *de novo* using the MEGAHIT assembler and compared against all viral sequences in the NCBI GenBank database as of 22 June 2016 (4). A single 7.4-kb-long contig was highly similar to the genome of Kunsagivirus A, with 50.8% pairwise identity across the coding sequence when aligned using ClustalW with an IUB cost matrix (gap extension cost, 6.66; gap open cost, 15). The novel virus, which we name Bakunsa virus (BKUV [sigil for baboon kunsagivirus]), probably represents a second species in the genus *Kunsagivirus*.

Our reconstruction of the coding-complete BKUV genome from a blood sample suggests that wild baboons in Africa are a natural host for kunsagiviruses. However, the absence of kunsagivirus sequences in other metagenomic studies of African monkeys (3, 5–11) indicates that these infections may be either acute or relatively rare if persistent. If kunsagivirus A truly infects birds, our discovery of a baboon kunsagivirus infers a broad host range for kunsagiviruses relative to members of other picornaviral genera. However, whether primates serve as the natural reservoir for some kunsagiviruses, or are an incidental “dead-end” host, remains an open question, and the natural course, incidence, and pathogenesis of kunsagivirus infections in baboons, or the potential of kunsagivirus cross-species transmission, remain unknown.

### Accession number(s).

The GenBank accession number of BKUV isolate baboon/M27-KuV/1986/TAN is KY670597.
